# Allergic rhinitis and other comorbidities associated with asthma control in Thailand

**DOI:** 10.3389/fmed.2023.1308390

**Published:** 2024-01-11

**Authors:** Thitiwat Sriprasart, Narongkorn Saiphoklang, Theerasuk Kawamatawong, Watchara Boonsawat, Wat Mitthamsiri, Naricha Chirakalwasan, Chirawat Chiewchalermsri, Athipat Athipongarporn, Harutai Kamalaporn, Kumpol Kornthatchapong, Manaphol Kulpraneet, Mongkhon Sompornrattanaphan, Nittha Oer-Areemitr, Ticha Rerkpattanapipat, Santi Silairatana, Sarita Thawanaphong, Thanate Gaensan, Ketsupar Jirakran, Orapan Poachanukoon

**Affiliations:** ^1^Division of Pulmonary and Critical Care Medicine, Department of Internal Medicine, Faculty of Medicine, Chulalongkorn University, King Chulalongkorn Memorial Hospital, Thai Red Cross Society, Bangkok, Thailand; ^2^Division of Pulmonary and Critical Care Medicine, Department of Internal Medicine, Faculty of Medicine, Thammasat University, Pathum Thani, Thailand; ^3^Division of Pulmonary and Critical Care Medicine, Department of Internal Medicine, Faculty of Medicine, Ramathibodi Hospital, Mahidol University, Bangkok, Thailand; ^4^Department of Medicine, Faculty of Medicine, Khon Kaen University, Khon Kaen, Thailand; ^5^Allergy and Clinical Immunology Division, Department of Medicine, Phramongkutklao Hospital, Bangkok, Thailand; ^6^Excellence Center for Sleep Disorders, Thai Red Cross Society, Bangkok, Thailand; ^7^Department of Medicine, Panyananthaphikkhu Chonprathan Medical Center, Srinakharinwirot University, Nonthaburi, Thailand; ^8^Department of Pediatrics, Phra Nakhon Si Ayutthaya Hospital, Phra Nakhon Si Ayutthaya, Thailand; ^9^Division of Pediatric Pulmonology, Department of Pediatrics, Faculty of Medicine, Ramathibodi Hospital, Mahidol University, Bangkok, Thailand; ^10^Department of Emergency Medicine, Faculty of Medicine, Thammasat University, Pathum Thani, Thailand; ^11^Division of Pulmonary and Critical Care, Department of Medicine, Srinakarinwirot University, Bangkok, Thailand; ^12^Division of Allergy and Clinical Immunology, Department of Medicine, Faculty of Medicine Siriraj Hospital, Mahidol University, Bangkok, Thailand; ^13^Division of Pulmonary and Critical Care Medicine, Ekachai Hospital, Samut Sakhon, Thailand; ^14^Allergy Immunology and Rheumatology Division, Department of Medicine, Faculty of Medicine, Ramathibodi Hospital, Mahidol University, Bangkok, Thailand; ^15^Division of Pulmonary Medicine and Pulmonary Critical Care, Department of Medicine, Faculty of Medicine Vajira Hospital, Navamindradhiraj University, Bangkok, Thailand; ^16^Department of Family Medicine, Faculty of Medicine, Ramathibodi Hospital, Mahidol University, Bangkok, Thailand; ^17^Center of Excellence for Maximizing Children’s Developmental Potential, Department of Pediatrics, Faculty of Medicine, Chulalongkorn University, Bangkok, Thailand; ^18^Center of Excellence for Allergy, Asthma and Pulmonary Diseases, Thammasat University, Pathum Thani, Thailand

**Keywords:** ACT, asthma, asthma control test, allergic rhinitis, comorbidities, IgE, quality of life, Thailand

## Abstract

**Background:**

Asthma and allergic rhinitis (AR) can coexist and cause disabilities. This study aimed to assess the association between AR, asthma control, asthma-related quality of life, and other comorbidities.

**Methods:**

A cross-sectional study was conducted in adults with asthma in six hospitals in Thailand. The outcomes were association of asthma control assessed by the asthma control test (ACT), AR, and asthma comorbidities. Not-well-controlled asthma was defined as ACT scores ≤22. The severity of AR was determined by visual analog scale (VAS). Severe AR was defined as VAS ≥5. Asthma-related quality of life (AQLQ), comorbidities, and total IgE were recorded.

**Results:**

A total of 682 asthmatic patients were included. Median (IQR) age was 58.0 (47.0–64.0) years. 69.9% were female. Not-well-controlled asthma was present in 44.7%. The prevalence of AR was 86.1%. Moderate/severe persistent AR was diagnosed in 21.7% and severe AR was diagnosed in 30.2% of the patients. Inhaled corticosteroid-containing regimens were prescribed in 97.7% of patients. Intranasal corticosteroid and antihistamine were prescribed in 65.7 and 31.7%, respectively. Patients with not-well-controlled asthma had higher body mass index, VAS scores, proportions of pollution exposure, aeroallergen sensitization, severe AR, nasal polyp, urticaria, food allergy, gastroesophageal reflux disease, depression and anxiety, peptic ulcer, and asthma exacerbations, but younger age, lower AQLQ scores, and lower FEV_1_. Correlation was found between AR severity and ACT (*r* = −0.461, *p* < 0.001), AQLQ (*r* = −0.512, *p* < 0.001), and total IgE (*r* = 0.246, *p* < 0.023). Multiple regression analysis revealed that ACT, AQLQ, and percentage of FEV_1_/FVC were significantly associated with severe AR.

**Conclusion:**

Allergic rhinitis is prevalent in Thai asthmatic patients. AR severity is associated with asthma control, quality of life, and pulmonary function. Comprehensive care is essential for patients with uncontrolled asthma, particularly when coexisting with conditions.

## 1 Introduction

Asthma is a heterogeneous disease, usually characterized by chronic airway inflammation. It is defined by the history of respiratory symptoms, such as wheeze, shortness of breath, chest tightness and cough that vary over time and in intensity, together with variable expiratory airflow limitation (1). Asthma and allergic rhinitis (AR) are characterized by chronic airway inflammation and components of other diseases. Asthma presents with variable respiratory symptoms and airflow limitation (2). AR presents with nasal congestion, sneezing, running nose, and ocular itch, which affect the patient’s quality of life (3). The majority of asthmatic patients have pulmonary and extrapulmonary comorbidities. AR, a common comorbidity in patients with asthma, affects asthma outcomes (3, 4). The prevalence of AR in patients with and without asthma in the Asia Pacific region varies according to the definition and recruited population (5–11). In a systematic review, the worldwide prevalence of AR is 18.1% (ranging from 1.0 to 54.5%) (12). Prevalence of AR ranged from 3.5 to 54.5% for America, from 1.0 to 43.9% for Europe, and from 1.0 to 47.9% for Asia (12). AR and other asthma-related comorbidities increase clinical complexities and healthcare costs (11, 13). Despite recommendations of asthma management and effective medications, particularly inhaled corticosteroids (ICS), a substantial number of asthmatic patients have not controlled their diseases. Ultimately, not-well-controlled asthma affects patient quality of life and leads to exacerbations and hospitalizations (13, 14).

An epidemiological survey of physician management of AR coexisting with asthma in the Asia Pacific region including Thailand revealed many problems (14, 15). Pulmonary and extrapulmonary comorbidities with asthma have been addressed for several decades (16). Obesity, obstructive sleep apnea (OSA), and gastroesophageal reflux disease (GERD) coexist in asthmatic patients (16–19). Comorbidities, including AR, contribute to risk factors and share inflammatory pathways with asthma. Both AR and asthma are inflammatory diseases and their inflammatory mechanisms are similar in that they are characterized by an inflammatory infiltrate made up of eosinophils, T cells, and mast cells that release several mediators, chemokines and cytokines, local and systemic IgE synthesis, and a systemic link via the bone marrow (20). These conditions drive the development of asthma and aggravate asthma control. Global and national asthma management guidelines recommend appropriate diagnosis and management of comorbidities. The interaction of asthma control, comorbidities, and health-related quality of life has never been systematically explored.

The aim of this study was to assess the association between AR and asthma control in adults in six medical centers in Bangkok and other provinces of Thailand. In addition, asthma-related quality of life and associations between asthma control and other comorbidities were assessed.

## 2 Methods

### 2.1 Study design and subjects

A cross-sectional study was conducted in asthma outpatient clinics from six tertiary medical centers in Thailand between December 2021 and December 2022. Consecutive asthmatic patients, aged 18 years or older and diagnosed according to the Global Initiative for Asthma strategy, were recruited from six university teaching hospitals.

Ethic approval for this study was obtained from the institutional committee on human research in each site of patient recruitment, in full compliance with international guidelines such as the Declaration of Helsinki, the Belmont Report, CIOMS Guidelines, and the International Conference on Harmonization-Good Clinical Practice (ICH-GCP). All methods were performed in accordance with these guidelines and regulations. All participants provided written informed consent.

### 2.2 Data collection

Allergic rhinitis was diagnosed by a clinician and by International Study of Asthma and Allergies in Childhood (ISAAC)-based questionnaires inquiring about the prevalence of AR and ocular symptoms (5, 8). The diagnosis of AR was made when the history and physical findings were consistent with an allergic cause (e.g., clear rhinorrhea, pale discoloration of nasal mucosa, and red and watery eyes) and one or more of the following symptoms: nasal congestion, runny nose, itchy nose, or sneezing. The severity of AR was assessed by a visual analog scale (VAS) ranging from 0 (no symptoms) to 10 (very severe symptoms) (3). Severe AR was defined as VAS ≥5.

Patient demographics and clinical characteristics including age, sex, age at initial asthma diagnosis, body mass index (BMI), smoking history, and indoor biomass and pollution exposure were recorded. Asthma exacerbations requiring emergency department (ED) visits in the past year and exacerbations requiring hospitalization in the past year were recorded. Current pharmacological and non-pharmacological treatments including bronchial thermoplasty were reviewed. Asthma medications including ICS, long-acting beta2-agonists, long-acting muscarinic antagonists, oral theophylline, type 2 biologics, and oral low-dose prednisolone, and AR medications including intranasal corticosteroid and oral medications were recorded. The daily dose of ICS was expressed as the budesonide equivalent in micrograms per day (2). Asthmatic patients were divided into elderly (65 years or older) and younger groups (younger than 65 years).

The Thai version of the Asthma Control Test (ACT™; scores from 5 to 25) was used to assess asthma control levels. Not-well-controlled asthma was defined as ACT ≤22 and uncontrolled asthma was defined as ACT ≤19 (21, 22). The Asthma Quality of Life Questionnaire (AQLQ) Thai version, 15-items with seven-point scale (scores from 15 to 105), was used to assess the asthma-related quality of life of patients (23, 24). Higher AQLQ scores indicated better quality of life. AR-related quality of life was assessed by the Rhinoconjunctivitis QoL Questionnaire (Rcq-36); 36 questions covering six domains (25).

Comorbidities including chronic rhinosinusitis, nasal polyps, OSA diagnosed by polysomnography, peptic ulcer, food allergy, aspirin hypersensitivity, atopic dermatitis, urticaria, diabetes, hypertension, obesity, coronary artery disease, and chronic kidney disease were assessed in the asthmatic patients and recorded. Disease-specific questionnaires for specific comorbidities were examined. Anxiety and depression were assessed using the Hospital Anxiety Depression Scale (HADS) questionnaire with subscales for both anxiety (HADS-A) and depression (HADS-D); scores ≥8 and ≥ 8 indicating anxiety and depression, respectively (26, 27). GERD was assessed by the Gastroesophageal Reflux Questionnaire (GerdQ); scores >9 suggesting GERD (28). A modified STOP-Bang questionnaire was used for assessing the risk of OSA; scores ≥3 suggesting risk for sleep apnea (29).

Spirometry was performed according to the American Thoracic Society and European Respiratory Society Standardization (30). Bronchodilator (BD) response test was done using salbutamol inhalation (total dose 400 μg) and repeating spirometry 15 min later. Pre-BD and post-BD forced expiratory volume in 1 s (FEV_1_), forced vital capacity (FVC), and FEV_1_/FVC were recorded and expressed in liters (L), %predicted, or %. Complete blood counts including eosinophil counts, serum specific IgE and total IgE were recorded.

### 2.3 Statistics analysis

We hypothesized that the prevalence of severe AR in patients with not-well-controlled asthma in our study would be 50%. The sample size was calculated for a prevalence survey using 5% margin of error, 80% power, and a two-sided alpha of 0.05. Therefore, the estimated sample size would be 385. Data were expressed as number (%), mean ± standard deviation or median (interquartile range; IQR). Chi-squared test was used to compare categorical variables between the two groups. Independent sample *t*-test was used to compare continuous variables with normal distributions between the two groups. The Mann–Whitney U test was used to compare between two independent groups on continuous variables with non-normal distributions. Pearson correlation was used for assessing the correlation between two independent continuous variables and presented as correlation coefficient (R). To determine factors associated with severe AR, we employed the logistic regression model with severe AR as the dependent variable. All independent variables—ACT, AQLQ, HADS-A, HADS-D, FEV_1_, FEV_1_/FVC, and total IgE—were entered into the regression model, followed by backward selection using a *p* value cutoff of 0.1. We report the regression coefficients along with 95% confidence interval, and corresponding *p* values. A two-sided value of *p* < 0.05 was considered statistically significant. Statistical analyses were performed using SPSS version 25.0 software (IBM Corp., Armonk, NY, United States).

## 3 Results

Six hundred eighty-two patients with asthma were included. Median (IQR) age was 58.0 (47.0–64.0) years. 69.8% were female. Pre-BD FEV_1_ was 80.00 (67.00–91.47) %. Blood eosinophil counts were 227.70 (100.00–437.51) cells/μL. Aeroallergen sensitization was 20.7%. ACT was 23.00 (20.00–24.00). The asthma was controlled in 55.3% of patients whereas in 44.7% of patients it was not well-controlled. 13.8% of patients had history of asthma exacerbations. 97.7% of patients received regular ICS treatment (Table 1).

**Table 1:** Baseline characteristics of asthmatic patients.

Characteristics	Data (*n* = 682)
Age, years	58.0 (47.0–64.0)
Male/Female	205 (30.1)/477 (69.9)
Body mass index, kg/m^2^	24.8 (22.4–28.1)
Age at initial asthma diagnosis ≤40 years	398 (58.4)
Smoking history	97 (14.2)
Indoor biomass exposure	126 (18.5)
Pre-BD FEV_1,_ L	1.99 (1.53–2.53)
Pre-BD FEV_1_, % predicted	80.00 (67.00–91.47)
Pre-BD FEV_1_/FVC, %	73.77 (64.49–81.39)
Blood eosinophil, cells/μL	227.70 (100.00–437.51)
Serum total IgE, IU/mL	155.00 (77.02–505.62)
Aeroallergen sensitization^a^	141 (20.7)
AQLQ, total scores	97.00 (83.00–105.00)
ACT, scores	23.00 (20.00–24.00)
Controlled asthma^b^	377 (55.3)
Not-well-controlled asthma^c^	305 (44.7)
History of asthma exacerbations	94 (13.8)
Exacerbation requiring ED visit in the past year	54 (7.9)
Exacerbation requiring hospitalization in the past year	17 (2.5)
ICS-containing therapy^d^	
No ICS or Intermittent ICS/formoterol	16 (2.3)
Regular low-dose ICS	315 (46.2)
Regular low-medium dose ICS	22 (3.2)
Regular medium ICS	201 (29.5)
Regular high-dose ICS	128 (18.8)
Add-on therapy	
LABA	632 (92.7)
LTRA	250 (36.7)
LAMA	112 (16.4)
Theophylline	45 (6.6)
Prednisolone	39 (5.7)
Bronchial thermoplasty	20 (2.9)
Type 2 biologics	7 (1.0)

Data shown as *n* (%) or median (IQR). ^a^Defined as positive skin prick test or specific IgE. ^b^Defined as ACT scores > 22. ^c^Defined as ACT scores ≤ 22. ^d^Daily ICS dose as budesonide equivalent (μg/day); low = 200–400, medium > 400–800, and high > 800. ACT, Asthma Control Test; AQLQ, Asthma Quality of Life Questionnaire; BD, Bronchodilator; ED, Emergency department; FEV_1_, Forced expiratory volume in 1 s; FVC, Forced expiratory volume; ICS, Inhaled corticosteroid; IgE, Immunoglobulin E; kg, Kilogram; L, Liter; LABA, Long-acting beta2-agonist; LAMA, Long-acting muscarinic antagonist; LTRA, Leukotriene receptor antagonist; m, Meter.

The prevalence of ISAAC-defined AR was 86.1%. Moderate/severe persistent AR was in 21.7% and severe AR was in 30.2% of patients. Intranasal corticosteroid and antihistamine were prescribed for AR treatment in 65.7 and 31.7% of patients (Table 2). Common comorbidities included hypertension (26.2%) and diabetes (10.7%), while the prevalence of coronary artery disease and chronic kidney disease was 2.8 and 2.5%, respectively. OSA was diagnosed in 12.0%, while STOP-Bang scores suggested a risk for sleep apnea in 11.0%. GERD was diagnosed by a clinician in 21.7% and by GerdQ in 1.9% (Table 2).

**Table 2:** Characteristics of allergic rhinitis and other asthma-related comorbidities.

Characteristics	Data (*n* = 682)
Allergic rhinitis	
AR^a^	540 (79.2)
AR by ISAAC criteria	587 (86.1)
ISAAC questionnaire: nose symptoms ever	577 (84.6)
ISAAC questionnaire: nose symptoms within 12 months	487 (71.4)
ISAAC questionnaire: nose with eye symptoms	285 (41.8)
ISAAC questionnaire: symptoms interfere with daily activity	275 (40.3)
Moderate/severe persistent AR by ARIA criteria	148 (21.7)
Severe AR^b^	206 (30.2)
VAS for AR severity, scores	2.0 (0–5.0)
INCS for AR treatment	448 (65.7)
Oral antihistamine for AR treatment	216 (31.7)
Comorbidities	
Chronic rhinosinusitis^a^	93 (13.6)
Nasal polyps^a^	48 (7.0)
OSA	82 (12.0)
OSA treated with CPAP	48 (7.0)
Risk of OSA^c^	75 (11.0)
GERD^a^	148 (21.7)
GERD diagnosed by GerdQ^d^	13 (1.9)
History of peptic ulcer	24 (3.5)
Depression and anxiety^a^	38 (5.6)
Depression by HADS screening	4 (0.6)
Anxiety by HADS screening	6 (0.9)
Food allergy^a^	71 (10.4)
Aspirin hypersensitivity^a^	51 (7.5)
Atopic dermatitis^a^	83 (12.2)
Urticaria^a^	109 (16.0)
Diabetes	73 (10.7)
Hypertension	179 (26.2)
Obesity	46 (6.7)
Coronary artery disease	19 (2.8)
Chronic kidney disease	17 (2.5)

Data shown as n (%) or median (IQR). ^a^Diagnosis by clinician. ^b^Defined as visual analog scale ≥ 5 points. ^c^Defined as STOP-Bang scores ≥ 3 points. ^d^Defined as GerdQ > 9 points. AR, Allergic rhinitis; ARIA, Allergic rhinitis and its impact on asthma; CPAP, Continuous positive airway pressure; GERD, Gastroesophageal reflux disease; GerdQ, Gastroesophageal reflux disease questionnaire; HADS, Hospital anxiety and depression scale; INCS, Intranasal corticosteroids; ISAAC, International Study of Asthma and Allergies in Childhood; and OSA, Obstructive sleep apnea.

Patients with not-well-controlled asthma had significantly higher BMI, higher VAS scores for AR severity, and higher proportions of indoor pollution exposure, aeroallergen sensitization, severe AR, nasal polyp, urticaria, food allergy, GERD, depression and anxiety, peptic ulcer, and asthma exacerbations, but they were younger and they had lower total AQLQ scores and FEV_1_ than patients with controlled asthma (Table 3). Moreover, a higher proportion of patients with not-well-controlled asthma had medium- to high-dose ICS treatment (Table 3).

**Table 3:** Factors associated with controlled asthma.

Variables	Controlled asthma^*^ (*n* = 377)	Not well-controlled asthma^*^ (*n* = 305)	*p* value
Age, years	58.0 (49.0–63.0)	55.0 (43.0–65.0)	0.018
Female sex	252 (67.0)	224 (73.4)	0.069
Body mass index, kg/m^2^	24.6 (22.3–27.3)	25.0 (22.5–29.4)	0.028
Age at asthma diagnosis, years	38.0 (22.3–50.0)	35.0 (17.0–48.0)	0.081
Smoking history	53 (14.2)	44 (14.5)	0.897
Indoor pollution exposure	57 (15.1)	69 (22.6)	0.012
Pre-BD FEV_1,_ %predicted	81.0 (70.0–92.0)	77.0 (64.0–90.3)	0.006
Pre-BD FEV_1_/FVC, %	73.8 (65.5–81.2)	73.3 (64.0–81.6)	0.843
Blood eosinophil, cells/μL	228.50 (120.70–416.00)	204.34 (73.15–472.20)	0.280
Serum total IgE, IU/mL	114.00 (44.20–286.00)	170.00 (110.76–557.00)	0.054
Aeroallergen sensitization	58 (15.4)	83 (27.2)	<0.001
AQLQ, total scores	105.00 (98.00–105.00)	83.50 (70.00–95.00)	<0.001
AR^a^	300 (80.0)	240 (79.2)	0.799
VAS for AR severity, points	1.00 (0.00–2.00)	3.00 (1.00–5.00)	<0.001
Severe AR	62 (16.4)	144 (47.2)	<0.001
Nasal polyp	18 (4.8)	30 (9.8)	0.010
Urticaria	45 (12.0)	64 (21.0)	0.001
Food allergy	27 (7.2)	44 (14.4)	0.002
GERD^a^	68 (18.1)	80 (26.2)	0.010
Depression and anxiety^a^	12 (3.2)	26 (8.6)	0.002
History of peptic ulcer	8 (2.1)	16 (5.3)	0.027
History of asthma exacerbations	19 (5.1)	75 (24.8)	<0.001
OSA	39 (10.4)	43 (14.1)	0.137
INCS for AR treatment	243 (64.5)	205 (67.2)	0.451
Medium to high-dose ICS for asthma treatment	133 (35.3)	196 (64.3)	<0.001

Data shown as *n* (%) or median (IQR). ^*^Controlled asthma defined as ACT > 22, whereas not well-controlled asthma defined as ACT ≤ 22. ^a^Diagnosis by clinician. ACT, Asthma Control Test; AQLQ, Asthma Quality of Life Questionnaire; AR, Allergic rhinitis; BD, Bronchodilator; FEV1, Forced expiratory volume in 1 s; FVC, Forced expiratory volume; GERD, Gastroesophageal reflux disease; ICS, Inhaled corticosteroid; IgE, Immunoglobulin E; INCS, Intranasal corticosteroid; IU, International unit; kg, Kilogram; L, Liter; m, Meter; mL, Milliliter; OSA, Obstructive sleep apnea; VAS, Visual analog scale; and μL, Microliter.

Correlation was found between severity of AR and ACT (*r* = −0.461, *p* < 0.001), AQLQ (*r* = −0.512, *p* < 0.001), HADS-A scores (*r* = 0.357, *p* = 0.006), HADS-D scores (*r* = 0.401, *p* = 0.002), and serum total IgE (*r* = 0.246, *p* < 0.023; Table 4). Spirometry parameters were not correlated to AR severity (Table 4). Multiple regression analysis revealed that ACT and AQLQ scores, along with percentage of FEV_1_/FVC, were significantly associated with severe AR (Table 5).

**Table 4:** Correlation between severity of allergic rhinitis and asthma characteristics.

Parameters	Severity of allergic rhinitis (VAS scores)	
	*R*	*p* value
ACT scores	−0.461	<0.001
AQLQ total scores	−0.512	<0.001
AQLQ symptom	−0.487	<0.001
AQLQ activity	−0.355	<0.001
AQLQ emotion	−0.427	<0.001
AQLQ environment	−0.453	<0.001
HADS anxiety subscale	0.357	0.006
HADS depression subscale	0.401	0.002
Pre-BD FEV_1_ (% predicted)	−0.037	0.368
Pre-BD FEV_1_/FVC (%)	0.020	0.635
Total IgE, IU/mL	0.246	0.023

ACT, Asthma Control Test; AQLQ, Asthma Quality of Life Questionnaire; BD, Bronchodilator; FEV_1_, Forced expiratory volume in 1 s; FVC, Forced expiratory volume; HADS, Hospital anxiety and depression scale; IgE, Immunoglobulin E; IU, International unit; mL, Milliliter; and VAS, Visual analog scale.

**Table 5:** Multiple logistic regression analysis for factors associated with severe allergic rhinitis in patients with asthma.

Variables	Regression coefficients	95% CI of coefficients	*p* value
Intercept	1.359	0.439, 2.279	0.009
ACT scores	0.553	0.028, 0.107	0.004
AQLQ scores	−0.898	−0.035, −0.016	<0.001
Pre-BD FEV_1_/FVC (%)	−0.672	−0.012, −0.005	0.001

ACT, Asthma Control Test; AQLQ, Asthma Quality of Life Questionnaire; BD, Bronchodilator; FEV_1_, Forced expiratory volume in 1 s; and FVC, Forced expiratory volume.

Compared to male patients, female patients exhibited lower BMI, AQLQ scores, blood eosinophil level, and proportions of smoking history, coronary artery disease, OSA, and use of medium to high-dose ICS treatment. However, they showed higher proportions of aeroallergen sensitization, severe AR and urticaria, as well as better pulmonary functions (Table 6).

**Table 6:** Differences in clinical characteristics between females and males with asthma.

Variables	Female (*n* = 477)	Male (*n* = 205)	*p* value
Age, years	57.0 (45.0–63.5)	58.0 (48.5–64.0)	0.208
Body mass index, kg/m^2^	24.5 (22.0–27.9)	25.2 (23.0–28.9)	0.015
Smoking history	19 (4.0)	78 (38.0)	<0.001
Indoor pollution exposure	88 (18.4)	38 (18.5)	0.978
Age at asthma diagnosis, years	36.0 (21.0–49.0)	38.0 (20.0–50.0)	0.869
Controlled asthma^*^	253 (53.0)	124 (60.5)	0.073
Pre-BD FEV_1,_ %predicted	81.0 (68.0–92.2)	78.0 (63.4–90.5)	0.042
Pre-BD FEV_1_/FVC, %	75.6 (66.1–83.0)	70.6 (59.9–78.0)	<0.001
Blood eosinophil, cells/μL	190.06 (80.56–437.47)	285.30 (160.80–451.82)	0.005
Serum total IgE, IU/mL	147.00 (51.45–505.62)	245.00 (134.00–519.00)	0.091
Aeroallergen sensitization	110 (23.1)	31 (15.1)	0.019
AQLQ, total scores	96.00 (81.00–105.00)	101.00 (91.00–105.00)	<0.001
AR^a^	380 (79.7)	160 (78.0)	0.726
VAS for AR severity, points	2.00 (0.00–5.00)	2.00 (0.00–4.00)	0.200
Severe AR	155 (32.5)	51 (24.9)	0.034
Nasal polyp	31 (6.5)	17 (8.3)	0.405
Urticaria	92 (19.3)	17 (8.4)	<0.001
Food allergy	51 (10.7)	20 (9.8)	0.728
GERD^a^	107 (22.5)	41 (20.0)	0.472
Depression and anxiety^a^	30 (6.3)	8 (3.9)	0.209
History of peptic ulcer	20 (4.2)	4 (2.0)	0.146
Diabetes	47 (9.9)	26 (12.7)	0.281
Hypertension	122 (25.6)	57 (27.8)	0.544
Coronary artery disease	9 (1.9)	10 (4.9)	0.030
Chronic kidney disease	9 (1.9)	8 (3.9)	0.117
Obesity	28 (5.9)	18 (8.8)	0.169
History of asthma exacerbations	73 (15.4)	21 (10.2)	0.073
OSA	47 (9.9)	35 (17.1)	0.008
INCS for AR treatment	312 (65.4)	136 (66.3)	0.814
Medium to high-dose ICS for asthma treatment	212 (44.4)	117 (57.1)	0.002

Data shown as *n* (%) or median (IQR). ^*^Controlled asthma defined as ACT > 22, whereas not well-controlled asthma defined as ACT ≤ 22. ^a^Diagnosis by clinician. ACT, Asthma Control Test; AQLQ, Asthma Quality of Life Questionnaire; AR, Allergic rhinitis; BD, Bronchodilator; FEV_1_, Forced expiratory volume in 1 s; FVC, Forced expiratory volume; GERD, Gastroesophageal reflux disease; ICS, Inhaled corticosteroid; IgE, Immunoglobulin E; INCS, Intranasal corticosteroid; IU, International unit; kg, Kilogram; L, Liter; m, Meter; mL, Milliliter; OSA, Obstructive sleep apnea; VAS, Visual analog scale; and μL, Microliter.

Elderly patients had a later age at asthma diagnosis, higher proportions of GERD, diabetes, hypertension, coronary artery disease, and chronic kidney disease, but lower pulmonary functions and proportions of AR and INCS use than younger patients (Table 7).

**Table 7:** Differences in characteristics between younger and elderly patients with asthma.

Variables	Younger (*n* = 520)	Elderly (*n* = 162)	*p* value
Age, years	53.0 (43.0–59.0)	71.0 (68.0–75.3)	<0.001
Female sex	112 (69.1)	365 (70.2)	0.798
Body mass index, kg/m^2^	24.8 (22.4–28.3)	24.4 (22.6–27.4)	0.372
Smoking history	69 (13.4)	28 (17.4)	0.204
Indoor pollution exposure	88 (16.9)	38 (23.5)	0.061
Age at asthma diagnosis, years	30.0 (16.0–44.0)	51.0 (12.3–66.0)	<0.001
Controlled asthma*	292 (56.2)	85 (52.5)	0.410
Pre-BD FEV_1,_ %predicted	81.07 (69.00–93.00)	73.00 (62.00–87.00)	<0.001
Pre-BD FEV_1_/FVC, %	76.68 (67.00–83.00)	67.65 (57.75–73.99)	<0.001
Blood eosinophil, cells/μL	219.43 (84.05–443.35)	249.60 (121.53–426.72)	0.220
Serum total IgE, IU/mL	151.87 (67.85–443.25)	194.50 (82.35–679.00)	0.550
Aeroallergen sensitization	104 (20.0)	37 (22.8)	0.436
AQLQ, total scores	98.00 (83.00–105.00)	96.00 (85.00–103.00)	0.096
AR^a^	441 (85.3)	99 (61.5)	<0.001
VAS for AR severity, points	2.00 (0.00–5.00)	2.00 (0.00–4.50)	0.673
Severe AR	158 (30.4)	47 (29.0)	0.739
Nasal polyp	32 (6.2)	16 (9.9)	0.107
Urticaria	82 (15.8)	27 (16.9)	0.739
Food allergy	51 (9.8)	20 (12.3)	0.360
GERD^a^	96 (18.5)	52 (32.3)	<0.001
Depression and anxiety^a^	29 (5.6)	9 (5.6)	0.983
History of peptic ulcer	14 (2.7)	10 (6.2)	0.037
Diabetes	34 (6.6)	39 (24.2)	<0.001
Hypertension	95 (18.3)	84 (51.9)	<0.001
Coronary artery disease	10 (1.9)	9 (5.6)	0.015
Chronic kidney disease	7 (1.4)	10 (6.2)	0.001
Obesity	7 (4.3)	39 (7.5)	0.162
OSA	59 (11.4)	23 (14.2)	0.334
History of asthma exacerbations	77 (14.9)	17 (10.6)	0.175
INCS for AR treatment	369 (71.0)	79 (48.8)	<0.001
Medium to high-dose ICS for asthma treatment	242 (46.5)	87 (53.7)	0.111

Data shown as *n* (%) or median (IQR). ^*^Controlled asthma defined as ACT > 22, whereas not well-controlled asthma defined as ACT ≤ 22. ^a^Diagnosis by clinician.

ACT, Asthma Control Test; AQLQ, Asthma Quality of Life Questionnaire; AR, Allergic rhinitis; BD, Bronchodilator; FEV1, Forced expiratory volume in 1 s; FVC, Forced expiratory volume; GERD, Gastroesophageal reflux disease; ICS, Inhaled corticosteroid; IgE, Immunoglobulin E; INCS, Intranasal corticosteroid; IU, International unit; kg, Kilogram; L, Liter; m, Meter, mL, Milliliter; OSA, Obstructive sleep apnea; VAS, Visual analog scale; and μL, microliter.

## 4 Discussion

This is a large multicenter study of correlation between asthma control, AR, and asthma-related comorbidities in Thailand. Our study revealed that AR was a common comorbidity (79% by clinician diagnosis) with asthma in Thai patients. The occurrence of AR in asthmatic patients has been addressed in previous large-scale studies and in the Allergic Rhinitis and its Impact on Asthma (ARIA) guidelines (31–33). Our AR prevalence in asthmatic patients (79%) is higher than studies in Saudi Arabia (33%) (34), Iran (47%) (35), Japan (67%) (36), Italy (60%) (37), and France (66%) (38) but is lower than the United States (94%) (39). There was concordance between the ISAAC-definition of AR and clinical diagnoses, indicating that diagnosis by clinicians was as reliable as the standard questionnaire used in epidemiological surveys. The most common AR symptoms classified by the ISAAC questionnaire reports were “nose symptoms ever” and “nose symptoms within 12 months” due to perennial AR. The prevalence of eye symptoms in our study (42%) was double the prevalence in a previous study in Thailand by Vichyanond et al. (6). These results can be explained by increased physician awareness of rhinitis coexisting with asthma (15). In addition, climate change and air pollution in Thailand aggravate AR, asthma, and other chronic respiratory diseases (40).

The majority of Thai asthmatic patients had mild AR, according to the ARIA criteria and VAS scores for symptom severity. The prevalence of mild AR in our study was comparable to the prevalence in two other studies (41, 42). This may be because the majority of AR patients were already treated with either intranasal corticosteroid or antihistamine. A relationship between rhinitis severity according to ARIA classification and medical treatment for AR and asthma was shown in a previous study by Antonicelli et al. (43). The patients in our study had controlled asthma according to ACT scores and had well-preserved lung function. The frequency of asthma exacerbation in our study (8% ED visits) was much lower than the frequency in a previous survey by Boonsawat et al. (44) (22% ED visits) in which less inhaled corticosteroid-containing regimens were used. The inverse correlation between AR and spirometry in Thai asthmatic patients resembles previous studies. They demonstrated that lung function abnormalities detected by spirometry in AR patients without clinical symptoms of asthma are directly correlated to rhinitis severity (45, 46). The frequency and severity of AR increased with the severity of asthma (33). Moreover, our results demonstrated that lower lung function determined by FEV_1_ was associated with not-well-controlled asthma. The effects of AR treatment and asthma treatment on the severity of both diseases have been shown. The most commonly prescribed rhinitis medication in our Thai asthmatics was intranasal corticosteroid, which was comparable to Western world studies (42, 47).

Severe AR determined by VAS score affected asthma control measured by ACT and asthma-related quality of life measured by AQLQ in our study. Low scores of symptom, activity, emotion, and environment domains of AQLQ were negatively influenced by more severe rhinitis. Our study also showed moderate negative correlations between AR severity and AQLQ score. When focusing on each domain in AQLQ, moderate correlations were found in the symptoms, emotional function, and environmental exposure domains, while a weak correlation was found in the activity limitation domain. The correlation between AR severity and AQLQ is possibly an indirect effect from AR and asthma or possibly due to overlap in the quality-of-life questionnaire as some questions are not specific to asthma morbidities but could be due to AR or other comorbidities (9, 48). Our study showed significantly negative correlations between AR severity by VAS score and ACT scores. This finding was similar to a previous report by Acevedo-Prado et al. (49). They found significant correlation between asthma symptoms and rhinitis severity in asthmatic patients.

Our study revealed a predominant proportion of females (70%), potentially explained by hormonal effects. Estrogen contributes to the inflammatory pathway by stimulating hormone receptors on dendritic cells, mast cells, CD4+ T lymphocytes (Th2), and eosinophils, possibly contributing to a higher number of asthma exacerbations in females (50). This effect might explain the higher percentage of aeroallergen sensitization observed in female asthmatic patients in our study. Additionally, we observed more severe AR and urticaria in this group. These factors might contribute to a worse asthma-related quality of life in female asthmatic patients.

Our study showed that Thai asthmatic patients have type-2 high asthma with high blood eosinophil levels and allergy driven diseases. Serum total IgE level generally tends to be higher in patients with allergy diseases compared with non-allergic individuals (51). Weak correlation between AR severity and IgE level was demonstrated in our study. Our findings were similar to a previous large population-based study which showed correlation between increased total IgE levels and asthma but not correlation between IgE levels and AR (52).

Other asthma-related comorbidities were addressed in our study including atopic dermatitis, eczema, chronic rhinosinusitis, nasal polyps, and food allergies. However, non-allergic comorbidities, for instance GERD, and anxiety and depression were commonly seen in Thai asthmatic patients. Other conditions which affect asthma control, i.e., age, BMI, AR severity, indoor pollution exposure, aeroallergen sensitization, nasal polyp, urticaria, food allergy, GERD, depression and anxiety, peptic ulcer, asthma exacerbations, and lung function, need to be considered. However, under-dose of ICS treatment is pivotal for achieving asthma control. These findings emphasize the roles of multimorbidity in asthma, association with allergic diseases, and the symptom burden (53). The interaction between GERD, OSA, and asthma is robust and complex (54). AR has been shown to be associated and it increases the risk of developing OSA. The putative mechanisms are increased airway resistance from nasal blockage and reduced pharyngeal diameter from breathing through the mouth (54). The prevalence of OSA in Thai asthmatic patients (12%) in our study is lower than a meta-analysis (49%) by Kong et al. (55). It might be because Thai asthmatic patients have near normal BMI leading to lower risk of OSA. Thai patients with not-well-controlled asthma had higher BMI than those with well-controlled asthma. These complexities of asthma and related comorbidities must be properly managed in specific clinical contexts.

Other important comorbidities in asthma are depression and anxiety. These were not uncommon in Thai asthmatic and AR patients in our study. A previous study by Rodrigues et al. (56) demonstrated that moderate/severe AR correlated with anxiety and depression scores by HADS. Our study showed higher percentage of anxiety and depression (higher HADS scores) in patients with not-well-controlled asthma. Furthermore, there was a significantly positive correlation between AR severity by VAS scores and anxiety or depression scores.

The relationship between AR severity and asthma-related parameters supports the concept of united airway disease and the link between quality of life and mental disorders. When lower airway-related symptoms are observed in AR patients, careful evaluation is strongly encouraged (57). Careful clinical evaluation and monitoring of AR patients are essential for achieving asthma control. This has been addressed in global and national asthma recommendations, including in Thailand (2, 3, 58). The prevalence and the importance of AR coexisting with asthma and other comorbidities must be addressed.

This study has some limitations. Firstly, some patient data might be missing from the multicenter study, therefore some results might not be accurate. Secondly, this study was conducted during the COVID-19 pandemic. This situation might have affected clinical outcomes such as asthma exacerbation rates, which might be lower than expected due to preventions of respiratory infection (e.g., mask wearing). Thirdly, according to the study, patients were examined only once, leading to the possibility of informational bias. It is possible that during the examination, patients did not exhibit signs of AR, and there is no information about episodes of such comorbidity in the period preceding the examination. Fourthly, the study exclusively enrolled adults, excluding children. Therefore, the findings are limited in their applicability to pediatric patients with asthma. Lastly, the study recruited patients only from tertiary hospitals; hence, data on disease severity and special treatment might be biased in selection. A longer prospective study is needed to determine long-term correlations between asthma and asthma comorbidities, including allergic diseases. For future perspectives, we believe there are potential advancements in the management of asthma and allergic rhinitis comorbidities. Personalized medicine may lead to more targeted treatments for individuals with asthma and allergic rhinitis in general practice. Meanwhile, a more holistic approach to managing asthma and AR may emerge as comprehensive patient management. This approach involves not only symptom management but also addressing underlying causes and contributing factors.

## 5 Conclusion

Allergic rhinitis is prevalent in Thai asthmatic patients. AR severity is associated with asthma control, quality of life, and pulmonary function. Several other comorbidities and parameters are also related to asthma control. Comprehensive patient care, including assessment, treatment, monitoring, and environmental control, is essential for patients with uncontrolled asthma, particularly when coexisting with conditions such as obesity, severe AR, nasal polyp, urticaria, food allergy, GERD, peptic ulcer, and psychiatric problems.

## Data availability statement

The raw data supporting the conclusions of this article will be made available by the authors, without undue reservation.

## Ethics statement

The studies involving humans were approved by the institutional committee on human research in each site of patient recruitment, in full compliance with international guidelines such as the Declaration of Helsinki, the Belmont Report, CIOMS Guidelines, and the International Conference on Harmonization-Good Clinical Practice (ICH-GCP). The studies were conducted in accordance with the local legislation and institutional requirements. The participants provided their written informed consent to participate in this study.

## Author contributions

TS: Conceptualization, Data curation, Formal analysis, Funding acquisition, Methodology, Software, Supervision, Validation, Visualization, Writing – review & editing. NS: Conceptualization, Data curation, Formal analysis, Investigation, Methodology, Validation, Visualization, Writing – review & editing. TK: Conceptualization, Data curation, Investigation, Methodology, Validation, Visualization, Writing – original draft, Writing – review & editing. WB: Conceptualization, Data curation, Investigation, Supervision, Validation, Visualization, Writing – review & editing. WM: Conceptualization, Data curation, Methodology, Visualization, Writing – review & editing. NC: Conceptualization, Investigation, Methodology, Visualization, Writing – review & editing. CC: Conceptualization, Formal analysis, Investigation, Methodology, Validation, Visualization, Writing – review & editing. AA: Conceptualization, Formal analysis, Methodology, Validation, Visualization, Writing – review & editing. HK: Conceptualization, Validation, Visualization, Writing – review & editing. KK: Conceptualization, Supervision, Validation, Visualization, Writing – review & editing. MK: Conceptualization, Data curation, Investigation, Methodology, Validation, Visualization, Writing – review & editing. MS: Conceptualization, Validation, Visualization, Writing – review & editing. NO-A: Conceptualization, Validation, Visualization, Writing – review & editing. TR: Conceptualization, Validation, Visualization, Writing – review & editing. SS: Conceptualization, Validation, Visualization, Writing – review & editing. ST: Conceptualization, Data curation, Investigation, Project administration, Resources, Validation, Visualization, Writing – review & editing. TG: Conceptualization, Validation, Visualization, Writing – review & editing. KJ: Data curation, Formal analysis, Project administration, Software, Visualization, Writing – review & editing. OP: Conceptualization, Funding acquisition, Investigation, Methodology, Project administration, Supervision, Validation, Visualization, Writing – original draft, Writing – review & editing.
